# Fats influencing flowering: Pistil-derived lipids affect pollen tube growth and fertility in *Arabidopsis thaliana*

**DOI:** 10.1093/plphys/kiae341

**Published:** 2024-06-14

**Authors:** Maneesh Lingwan

**Affiliations:** Plant Physiology, American Society of Plant Biologists; Donald Danforth Plant Science Center, St. Louis, MO 63132, USA

In flowering plants, pollen germination and subsequent pollen tube growth is crucial for efficiently transporting nonmotile sperm cells to the egg and facilitating reproduction. These activities are carried out via specific remodeling of membrane systems and lipid metabolism. Successful pollen germination requires substantial quantities of phospholipid to rapidly synthesize and modify membranes during pollen growth and tube elongation. Disturbance of membrane lipids often results in reduced pollen transmission and male fertility (Ischebeck 2016).

**Figure 1.**
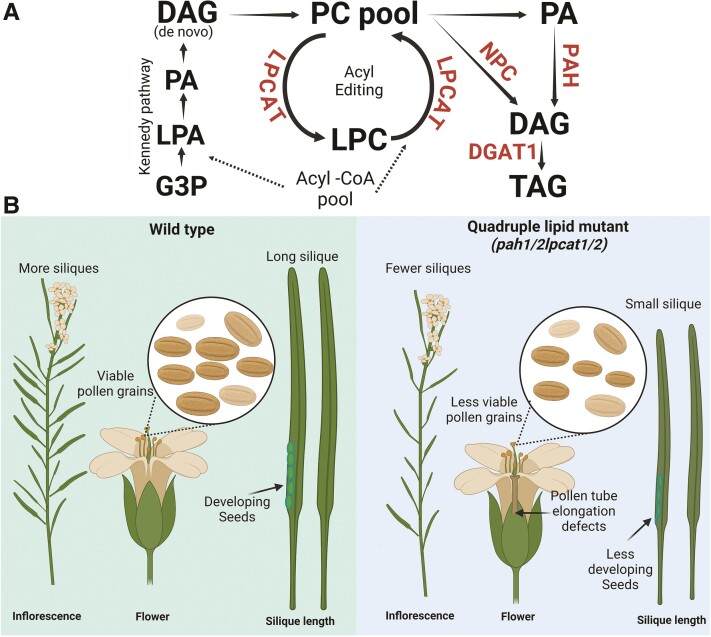
Illustration representing how disruption of lipid metabolism impacts reproductive phase of plant development. **A)** Pathway depicting major routes of PC biosynthesis and turnover through LPCAT, phosphatidic acid PAH, DGAT1, and NPC in lipid biosynthesis. **B)** Wild-type plants under normal growth conditions demonstrate abundant siliques, viable pollen grains, and long siliques. In contrast, deficiency of PAH and LPCAT compromises flower growth and pollen transmission. The knockout quadruple mutant *pah1/2 lpcat1/2* plants show fewer siliques, less viable pollen, less pollen tube growth, small siliques, and defects in developing seeds. The green and blue panels represent wild type and quadruple lipid mutants, respectively. Figure data were adapted from Song et al. (2024) and created using BioRender.

The most abundant nonplastidial membrane phospholipid is phosphatidylcholine (PC), which is critical in maintaining membrane lipids. PC is synthesized via multiple routes, but for de novo biosynthesis, diacylglycerol (DAG) is an essential precursor. The specialized enzyme phosphatidic acid phosphohydrolase (PAH) catalyzes DAG production, while lysophosphatidylcholine acyltransferase (LPCAT) transforms lysophosphatidylcholine (LPC) into PC (Fig. 1A). Previous studies showed that deficiencies of LPCAT accelerated PC turnover in Arabidopsis and impacted lipid metabolism (Wang et al. 2012, 2014). However, the evidence supporting membrane lipids and their impact on sexual reproduction is still limited and critical to understanding pollen development.

In this issue of *Plant Physiology*, Song et al. (2024) uncovered the combined roles of PAH and LPCAT deficiencies in PC metabolism and their impact on the reproductive phase of plant development. Knockout double mutants of either *lpcat1/2* or *pah1/2* had comparable phenotypes to wild type (WT) in silique length and produced close to 100% fertilized seeds. However, the quadruple mutant that combined deficiencies of *pah1/2* and *lpcat1/2* in had shorter siliques, aborted ovules, and reduced pollen transmission rate (Fig. 1B). Pollen viability assays showed that quadruple mutants have reduced viable pollen grains compared with WT. The observed effects are specific to the paternal line. When the quadruple mutant is the maternal parent and the paternal parent is WT, silique length and seed set were comparable with WT; but when the quadruple mutant is the paternal parent and the maternal parent is WT, seed set and silique length are comparable with the self-fertilized quadruple mutant.

Pistils are female reproductive organs that possess abundant glycerolipid lipids like DAG (Nakamura 2015). A comprehensive transcriptomics analysis indicated diacylglycerol acyltransferase (*DGAT1*) and non-specific phospholipase C (*NPC6*, *NPC2*) genes are highly expressed in the pistil and found to be essential for male gametophyte development (Mondragón-Palomino et al. 2017). DGAT1 catalyzes the conversion of DAG to triacylglycerol (TAG), which are major storage lipids in developing seeds (Yen et al. 2008). The authors hypothesized that the fertilization deficiency phenotype could be rescued by increasing the supply of DAG to the pollen tubes. The supply of DAG can impact other lipids on the pistil to enhance pollen performance; in the *dgat1* mutant, the pool of DAG levels was elevated up to 12% compared with the WT, while the *npc6-2* mutant showed the opposite phenotype and had decreased DAG formation capacity. When *pah1/2 lpcat1/2* pollen was used to fertilize the *dgat1* mutant, the fertilization rate was comparable with WT. When pollen from the quadruple mutant was used to fertilize *npc6* pistils, pollen tube growth and fertilization rates were even lower than those in the self-fertilized quadruple mutants (Song et al. 2024). These data show that DAG levels in the pistil can complement or exacerbate the impacts of the lipid defects in the pollen.

DAG is a critical precursor for phospholipid metabolism and is known to occupy a unique and well-connected position in the lipid metabolic network. Song et al. (2024) tested the effect of exogenous DAG supplementation in vitro on pollen elongation. Different molecular species of DAG at varied concentrations were supplemented in the form of liposomes, and in some cases they significantly affected pollen tube elongation. The fatty acids C16:0 and C18:1 appeared to be most efficient in restoring the phenotype of pollen tube growth in the *pah1/2 lpcat1/2* quadruple mutants. Fluorescence-labeled DAG (TopFluor) was applied in the in vitro system to test whether pollen tubes could take up DAG, and both WT and mutant pollen took up the fluorescent signal after germination. This finding was corroborated by thin layer chromatography, which showed a fluorescent band corresponding to PC and phosphatidylethanolamine. These results support the idea that exogenous DAG has positive effects on pollen tube growth and can be incorporated into phospholipid production.

Song et al. (2024) provide noteworthy insights into the involvement of lipid metabolism in the interactions between male and female reproductive structures in plants. Recent findings suggest that changing environmental conditions can impact pollen fertility and susceptibility of stigma tissue (Poulsen et al. 2015; Sah and Sofo 2024). In the future, it would be interesting to explore high-throughput multi-omics techniques to comprehensively understand lipid metabolism and the mechanisms of interactions between pollen tubes and pistils in plants, especially in abiotic stress conditions that can significantly impact crop yield by affecting fertility.
